# Genotype–Phenotype Correlation in a New Fabry-Disease-Causing Mutation

**DOI:** 10.3390/medicina55050122

**Published:** 2019-05-07

**Authors:** Agnė Čerkauskaitė, Rimantė Čerkauskienė, Marius Miglinas, Arvydas Laurinavičius, Can Ding, Arndt Rolfs, Lina Vencevičienė, Jūratė Barysienė, Edita Kazėnaitė, Eglė Sadauskienė

**Affiliations:** 1 Institute of Biomedical Sciences, Faculty of medicine, Vilnius University, Santariškių 2, 08406 Vilnius, Lithuania; Arvydas.Laurinavicius@vpc.lt (A.L.); Edita.Kazenaite@santa.lt (E.K.); 2 Institute of Clinical Medicine, Faculty of medicine, Vilnius University, Santariškių 2, 08406 Vilnius, Lithuania; rimante.cerkauskiene@gmail.com (R.Č.); Marius.Miglinas@santa.lt (M.M.); lina.venceviciene@mf.vu.lt (L.V.); Jurate.Barysiene@santa.lt (J.B.); Egle.Sadauskiene@santa.lt (E.S.); 3 Institute of Human Genetics, University Medical Center of the Johannes Gutenberg University Mainz, Langenbeckstraße 1, 55131 Mainz, Germany; dingcangut@gmail.com; 4 University of Rostock, Gehlsheimerstrasse 20, 18147 Rostock, Germany; arndt.rolfs@med.uni-rostock.de; 5 Centogene AG, Am Strande 7, 18055 Rostock, Germany

**Keywords:** Fabry disease, α-galactosidase A, *GLA* gene, novel mutation, classical manifestation

## Abstract

*Background*: Fabry disease (FD) is a rare X-linked inherited lysosomal storage disorder caused by α-galactosidase A deficiency leading to intracellular glycosphingolipid accumulation. FD manifestation is multisystem, and can differ depending on disease-related genetic variants. Currently, more than 700 different FD-causing mutations have been identified in the human *GLA* gene. We identified a novel mutation in a Lithuanian family with classical manifestations of Fabry disease, revealing severe effects to the cardiovascular systems of heterozygous women. *Case presentation*: A 49-year-old woman underwent echocardiography due to progressive dyspnea that lasted seven years, reduced physical activity, and periodic cardiac arrhythmia. Echocardiography revealed left ventricular hypertrophy with normal diastolic function. The patient had experienced acroparesthesia in her upper limbs and abdominal pain since childhood, and in the last decade had experienced mild proteinuria without renal failure. Her renal biopsy was typical for Fabry disease. The patient’s brain magnetic resonance imaging (MRI) (T2 flair) showed white matter hyperintensities lesions. DNA sequencing of the proband, her mother and one of her sons showed a novel *GLA* gene exon 2 mutation, c.270C>G (p.Cys90Trp). All three patients had decreased α-galactosidase A activity and specific FD manifestations. *Conclusions*: A novel *GLA* mutation, c.270C>G (p.Cys90Trp), was found in a Lithuanian family with a classical form of Fabry disease in heterozygous women with predominant cardiac involvement. However, the exact manifestation of this mutation is still unclear as it is newly reported and further research must be done.

## 1. Introduction

Fabry disease (FD, OMIM#301500) is a rare X-linked lysosomal storage disorder caused by α-galactosidase A deficiency leading to the progressive accumulation of glycosphingolipids with terminal α-D-galactosyl residue, specifically globotriaosylsphingosine (lyso-Gb3) and globotriaosylceramide (GL-3, Gb3) [1,2,3]. The storage of these lipids within the lysosomes of various body cells and organs results in severe and potentially life-threatening multisystem complications such as progressive hypertrophic cardiomyopathy and fibrosis, chronic kidney disease, peripheral neuropathy, stroke, and eventually death [4,5,6]. Classically described as affecting hemizygous males with undetectable or very low residual enzymatic activity, it is now known that this disease affects both genders, with later and less-severe manifestations in heterozygous females presumably because of random X chromosome inactivation [5,7]. As with many genetic diseases, the spectrum of phenotypes among patients with the same *GLA* mutation can vary widely [4,8]. According to previous reports, the classic form of Fabry disease starts in childhood or adolescence and usually includes symptoms such as angiokeratoma, acroparesthesia, abdominal pain, corneal opacities, hypohidrosis, hearing disorders, cardiac lesions, renal insufficiency, and stroke, leading to high morbidity and mortality [5,8,9]. Patients with variant forms typically present milder clinical manifestations, mostly limited to the heart or the kidneys. These comprise almost 70% of all FD cases [10]. Progressive hypertrophic cardiomyopathy and fibrosis can result in various cardiac complications, including heart failure, arrhythmias, conduction abnormalities, and atrial fibrillation. According to the Fabry Outcome Survey (FOS), cardiac arrest is one of the major cause of deaths in FD in both sexes [7,11,12].

Long-term studies showed that enzyme replacement therapy (ERT) can decelerate the progression of myocardial hypertrophy and the development of heart failure, if started prior to established myocardial fibrosis. Furthermore, ERT has the potential benefit of slowing the progression of kidney involvement in FD [5].

To date, more than 900 mutations have been identified in the human *GLA* gene as being disease-causing for FD, including missense mutations, small deletions/insertions, splice mutations, and large gene rearrangements [4,13,14]. All mutations were collected in the Human Gene Mutation Database and others (http://www.hgmd.cf.ac.uk/ac/gene.php?gene=GLA; http://fabry-database.org). However, there are still undescribed mutations in the *GLA* gene.

In this report, we present a novel *GLA* gene mutation in a Lithuanian family with classic FD phenotypes. We also discuss clinical features and the molecular diagnosis of FD.

## 2. Case Presentation

A 49-year-old woman was admitted to Vilnius University Hospital Santaros Klinikos cardiology unit due to progressive dyspnea, reduced physical activity, and periodic cardiac arrhythmia. A full cardiovascular examination was performed.

Analysis of medical history revealed that during childhood the patient suffered from acroparesthesia, heat intolerance, and severe abdominal pain with gastrointestinal abnormalities such as diarrhea and constipation. Bronchial asthma had been diagnosed and experienced since her adolescence. She had been followed by a nephrologist since the age of 20 because of recurrent pyelonephritis, acute urinary tract infections, subnephrotic proteinuria (<1 g/L) and edema in her lower limbs. Glomerulus filtration rate was normal. Kidney ultrasound showed no changes. At the age of 39 palpitations of the heart, dyspnea, reduced physical activity, and low blood pressure were noticed. At the same age, she manifested episodes of tinnitus, hearing impairments, and some angiokeratoma in the umbilical region.

During evaluation in our center, her electrocardiogram (ECG) showed normal sinus rhythm, short PR interval, as well as signs of left ventricular (LV) hypertrophy with secondary repolarization abnormalities. Echocardiography revealed asymmetric myocardial hypertrophy in the LV apex and in the lateral wall without LV outflow tract obstruction. Two-dimensional strain analysis showed a reduced global longitudinal strain (GLS−14%), especially reduced in the posterolateral wall (peak systolic strain−8%), whereas diastolic function of the LV was normal. Cardiac magnetic resonance imaging (MRI) also showed an asymmetric myocardial hypertrophy which was most significant in the apex and the lateral wall of the LV. Gadolinium enhancement imaging did not disclose any fibrotic alterations of the myocardium.

Given the clinical suspicion of Fabry disease, the patient underwent complete diagnostic workup which revealed specific ophthalmological findings (cornea verticillata) and mild proteinuria without signs of renal failure. Brain MRI on T2/FLAIR revealed white matter hyperintensities lesions while no other findings including ischemic or vascular abnormalities were observed. Color Doppler of the extracranial arteries showed normal carotid diameter with no occlusions or vasospasm. Electroneurography of upper and lower extremities revealed ordinary amplitude and conduction of motor nerves with no signs of polyneuropathy.

**Figure 1:**
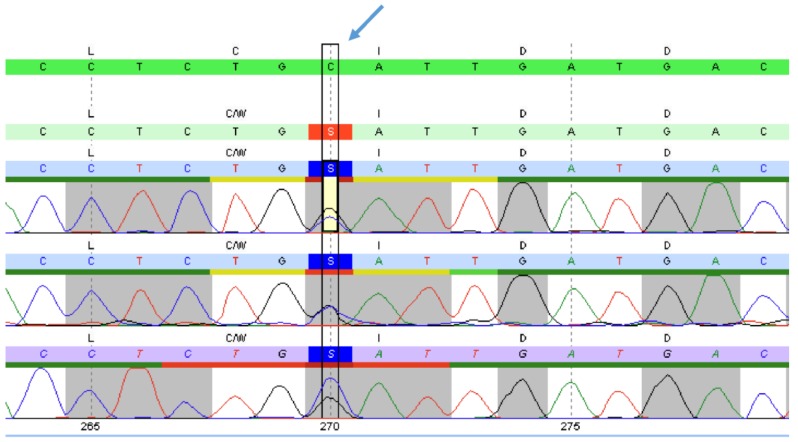
Alignment of gene sequences. Portion of the sequence of exon 2 of the *GLA* gene in the patient. The blue arrow indicates the position of the mutation c.270C>G (p.Cys90Trp).

Considering all clinical manifestations, Fabry disease was suspected and genetic and enzymatic analyses were thus performed. Peripheral blood samples were collected and DNA was extracted using the GenElute Blood Genomic DNA Kit (Sigma-Aldrich, USA). The *GLA* gene was analyzed by PCR and sequencing of the entire coding region and the highly conserved exon–intron splice junctions was performed. The concentration of the biomarker lyso-Gb3 in a dried blood spot was measured using HPLC and tandem mass spectrometry. The patient’s *GLA* gene sequencing analysis revealed a heterozygous mutation in exon 2 of GLA, c.270C>G (p. Cys90Trp). It is located in a weakly conserved nucleotide and highly conserved amino acid position, with large physicochemical differences between the exchanged amino acids (Alamut v.2.7.1) (Figure 1). The concentration of the biomarker lyso-Gb3 was pathologically increased to 10.0 ng/mL (reference: ≤1.8 ng/mL).

**Figure 2:**
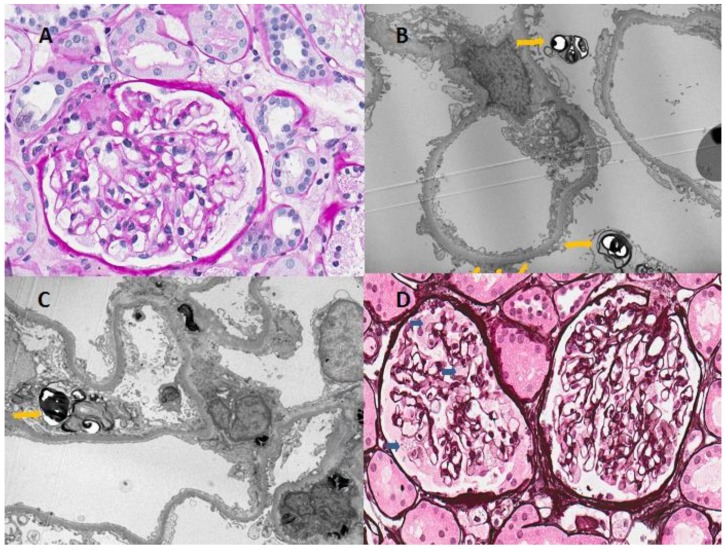
Light and electron microscopic findings of renal biopsy from the patient. (**A**) Microscopic examination revealed minimal mesangial proliferative changes; (**B**,**C**) Typical findings of Fabry disease-multilamellated myelin figures (yellow arrowhead) are seen in the cytoplasm of podocytes on electron microscopy; (**D**) Light microscopy showed the vacuolization of podocytes (blue arrowhead), although the patient had normal renal function and nonsignificant proteinuria (Haemotoxylin and Eosin stain, ×400).

Since a previously unreported mutation was detected, a kidney biopsy was performed on the proband to support the diagnosis, in which ultrastructural pathognomic changes consistent with Fabry disease were found (Figure 2). Light microscopy examination showed enlarged podocytes with foamy vacuoles. Electron microscopy identified typical electron-dense multilamellar inclusions and zebra bodies in the cytoplasm of podocytes as well as focal podocyte foot process effacement.

**Figure 3:**
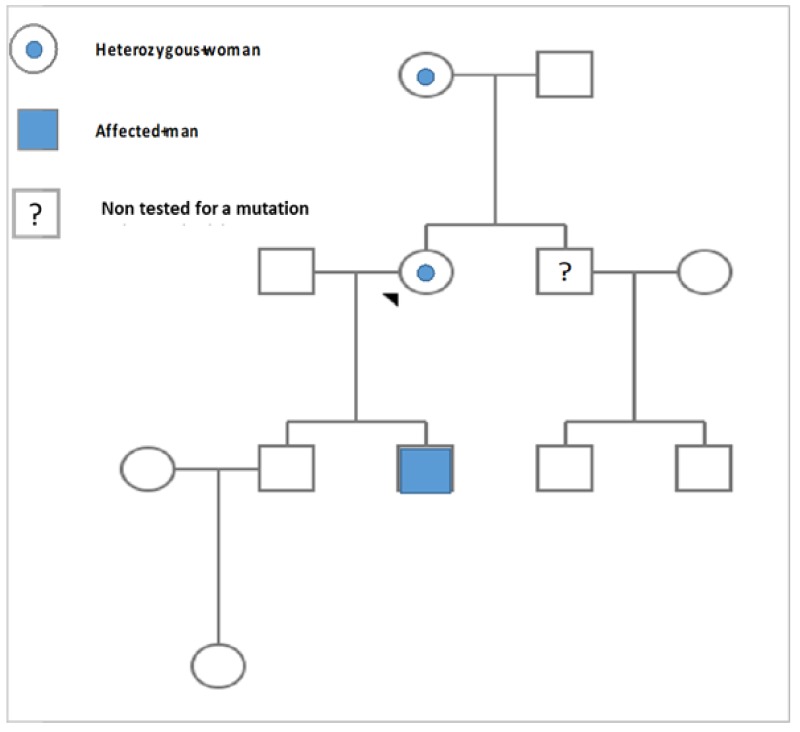
Pedigree of the family of the patient. The proband is a heterozygous carrier for her mutation, inherited from her mother. One of the patient’s sons is affected by Fabry disease, while the other son is healthy. The brother of the proband should also be screened.

Once the diagnosis of Fabry disease was confirmed, genetic analysis was extended to the family members of the patient (mother, two sons, and her brother) (Figure 3). The sequence analysis of the *GLA* gene showed that the patient’s 70-year-old mother was heterozygous for c.270C>G (p. Cys90Trp) mutation. The concentration of the lyso-Gb3 biomarker was pathologically increased (13.4 ng/mL). She was diagnosed with HCMP and suffered from heart failure. Since she had experienced syncope and life-threatening ventricular arrhythmia, a cardioverter-defibrillator had been implanted. Fabry disease was also confirmed in one of the patient’s sons by measurement of leukocyte α-galactosidase A activity (0.8 (limit of detection) µmol/L/h (reference ≥15.3 µmol/L/h)) and DNA analysis. The concentration of the biomarker lyso-Gb3 was significantly increased (98 ng/mL). The 25-year-old son revealed clinical manifestations referable to Fabry disease such as severe acroparesthesias, anhidrosis, heat intolerance, clustered angiokeratoma on the thighs, tinnitus, abdominal pain, diarrhea, as well as depression and specific ophthalmological findings (cornea verticillata). The results of the blood and urine analysis were in the normal range. His kidney ultrasound and brain MRI showed no changes. Echocardiography revealed heart morphology and function to be normal. Electrocardiography showed electrical left-ventricle hypertrophy (LVH).

Both the proband and her son were referred for ERT treatment a year following diagnosis. agalsidase alfa was given at 0.2 mg/kg body weight every other week by intravenous (IV) infusion. Regular assessments of the impact of ERT on all affected organ systems were performed according to Fabry disease recommendations [15]. The mother had significant improvements of her cardiac symptoms, while her son experienced marked clinical beneficial effects on acroparesthesias and all manifestations due to early diagnosis and management of Fabry disease according to the literature [16]. Better responses to treatment in decreasing lyso-Gb3 from baseline 92.1–39.7 to 30.4 ng/L (reference value <1.8 ng/L) after two years of initiating ERT was observed in the proband’s son compared with his mother (8.9 ng/L–8.7 to 10.5ng/L). The patient’s mother was not treated with ERT due to her age and late diagnosis, since there is no clinical evidence for its effectiveness, and the benefit for the elderly is doubtful in terms of life expectancy and cost effectiveness. Decision to treat can be influenced by advanced elderly age of the patients and severe comorbidities [15]. The summary of clinical and pathological findings and treatment of the patient and her family members is shown in Table 1.

## 3. Discussion

FD is a rare complex multisystem disease with high heterogeneity of its manifestations, causing various clinical symptoms in both heterozygous and hemizygous individuals.

In this paper, we report a new *GLA* gene mutation (c.270C>G (p. Cys90Trp) in one Lithuanian family with two different phenotypes: a predominantly cardiac variant in heterozygous women and a classic form of Fabry disease in a hemizygous man. This new mutation had not been previously reported in FD databases [17]. Defects in the *GLA* gene coding region can cause alterations in the amino acid sequence leading to changes of *GLA* conformation and enzyme dysfunction.

Primarily heterozygous females carrying the *GLA* mutation were asymptomatic for Fabry disease. Recent studies showed that skewed X chromosome inactivation has a significant impact on the phenotype and natural history of females with FD [18]. The degree and direction of skewed X chromosomes can lead to significant differences in the activity of α-galactosidase A in the body tissues, accounting for the differences in organ damage and its severity. In this report, our female patients had significant cardiac symptoms and mild proteinuria, which might be attributed to random X chromosome inactivation [19,20]. Heterozygous carriers with FD have high variability in the development of mild to severe clinical manifestations, which we also observed in the two female patients from the reported family. Enzymatic activity can be within normal range and the moderate accumulation of plasma lyso-Gb3 can be seen in 40%–60% of female patients [21]. This appears to be due to the chimerism of normal and affected cells, since one of the two X chromosomes in each somatic cell becomes randomly inactive during embryogenesis [21]. Therefore, genetic confirmation of Fabry disease and further genetic investigations including skewed X chromosome inactivation analysis becomes essential and should be performed in our female patients [22,23].

According to the Fabry Outcome Survey (FOS) database, cardiac manifestations including palpitations, syncope, dyspnea, or angina were reported in 69% of affected men and 65% of affected women, beginning at a mean age of 32.9 years in males and 36.4 years in females [12]. Our proband and her mother had significant cardiac symptoms including palpitations, dyspnea, and rhythm abnormalities. Atrial fibrillation, bradycardia, and conduction abnormalities are common findings in Fabry disease [24]. Chronic Gb3 accumulation in cardiomyocytes, endothelial cells, and vascular smooth muscle cells, valve fibroblasts, and conductive system cells causes dysfunction of the cells, interferes with intracellular signaling pathways, and consequently leads to hypertrophy, apoptosis, necrosis, and fibrosis. In this report, female patients presented left ventricular hypertrophy which is associated with a higher prevalence of cardiac symptoms and arrhythmias. Malignant arrhythmias are often associated with the presence of high-degree fibrosis followed by hypertrophic cardiomyopathy in male patients [25]. However, the significant myocardial hypertrophy and fibrosis causing the cardiac arrhythmia in the patient’s mother illustrates that female patients can also present with severe cardiac manifestations, and implantation of a cardioverter-defibrillator could be the only solution for these patients [5,26,27]. Overall, cardiac arrest is one of the most common causes of death in Fabry disease in both sexes [28,29,30]. Low blood pressure is also a typical sign of FD. Hypotension was observed in all three patients. This could be associated with FD itself or because of the use of antihypertensive agents such as renin angiotensin system blockers, which are recommended for their beneficial effect on the regression of myocardial hypertrophy and protection of the kidneys. Heart failure can be caused by many factors, and it should be treated according to general principles beyond ERT.

Dyspnea presentation in our female patients is a frequent symptom among FD patients. It may have many different etiologies, including disturbed diastolic and/or systolic cardiac functions or respiratory dysfunction. A registry study has recently reported that obstructive airway disease is up to 10 times more prevalent in FD patients compared to the general population [5]. Therefore, complete pulmonary tests allowing early detection of the signs of airway obstruction should also be considered as a useful screening tool for individuals with Fabry disease [31].

Proteinuria is a common factor related with progressive loss of kidney function in many forms of chronic kidney disease [32]. The proband presented mild, non-nephrotic proteinuria with normal renal function. Although most heterozygous carriers do not develop kidney disease with glycosphingolipid accumulation, some heterozygous females with FD may have typical findings in kidney biopsies [33]. Although the kidney specimen of the proband showed almost normal appearance under light-microscopy examination, the electron microscopy revealed lamellated myelin structures (zebra bodies), confirming the FD diagnosis. Therefore, kidney biopsies have both diagnostic and prognostic value for patients with FD. According to the literature, Gb3 deposits can been seen in most renal cell types at 17 weeks of gestation [5]. Studies of kidney biopsies have indicated the age-dependent progressive accumulation of glycosphingolipids, causing early damage of podocytes and subsequently leading to proteinuria [34]. Enlarged mesangial matrices with segmental or global glomerulosclerosis, interstitial fibrosis, tubular atrophy, and arteriosclerosis leading to decreased kidney function are also common findings in more advanced FD cases. The patient’s son had no renal manifestations, hence kidney biopsies were not performed. However, this procedure should definitely be considered in the future to evaluate the presence and severity of Fabry nephropathy as well as the progression of the disease and efficacy of the treatment [35]. In addition, although the affected son had no severe Fabry disease manifestations at the age of 26 years, severe Fabry manifestations such as cardiac involvement, cerebrovascular disease, nephropathy, and other organ damage with life-limiting effects can appear in any period of life and should be examined during regular follow up each year by a multidisciplinary team.

Moreover, all the patients reported in this paper are under agents of a renin angiotensin system blockade, as this antihypertensive drug is proposed to be the most effective treatment for the reduction of proteinuria and prevention of microalbuminuria [5].

Early diagnosis of FD is crucial for early instauration of the treatment and prevention of progressive multisystem organ failure due to the natural disease course. Approved standards of patient care therapies increase quality of life and ensure longer life expectancy. ERT is the first-line specific treatment, having a positive effect on stabilizing and improving kidney function and reducing the long-term progression of hypertrophic cardiomyopathy, especially if ERT is started prior to the development of established myocardial fibrosis (less than three segments) [5,36,37]. A recent study revealed that the incidence of rapid increases in left ventricular mass is three to four-fold higher in untreated patients when compared with the treated group [38]. Unfortunately, long-term follow up studies showed that ERT cannot prevent the progression of existing significant fibrosis and sudden cardiac death [37,39]. In addition, there is no reduction in the rate of stroke with ERT.

Further investigations of improved treatments in FD should be performed, including pharmacological chaperones, substrate reduction therapy, and modified α-galactosidase A with longer half-life and better tissue penetration.

## 4. Conclusions

In conclusion, we report a novel *GLA* mutation, c.270C>G (p.Cys90Trp) in exon 2, which was found in one Lithuanian family. Our study suggests the presence of a genotype–phenotype correlation for the c.270C>G (p.Cys90Trp) mutation in classical manifestations of Fabry disease, more severely affecting the cardiovascular systems of heterozygous women. However, the exact manifestation of this mutation is still unclear, as it is newly reported, and further research must be done.

## Figures and Tables

**Table 1:** Clinicopathological findings and treatment of the patient and her family members described in this case report.

Subject	Age of Diagnosis (Years)	Clinical Manifestation	p. Cys90Trp Mutation	Leukocyte α-Galactosidase A Activity (Reference ≥15.3 µmol/L/h)	^†^ ERT
Patient	49	Palpitations, dyspnea and rhythm abnormalities, LVH, mild proteinuria, angiokeratoma, tinnitus, gastrointestinal abnormalities, cornea verticillata, electrical LVH	Present	Not applicable	Started
Patient’s mother	70	Palpitations, dyspnea and rhythm abnormalities, LVH, mild proteinuria	Present	Not applicable	Not investigated
Patient’s son 1	25	Severe acroparesthesias, anhidrosis, clustered angiokeratoma on the thighs, tinnitus, abdominal pain, diarrhea, cornea verticillata, depression	Present	0.8 (limit of detection) µmol/L/h	Started
Patient’s son 2	28	Unremarkable	Not present	≥15.3 µmol/L/h	Not investigated

^†^ ERT: enzyme replacement therapy. LVH: left ventricular hypertrophy.
